# Involvement of Sirtuin 1 in the Growth Hormone/Insulin-like Growth Factor 1 Signal Transduction and Its Impact on Growth Processes in Children

**DOI:** 10.3390/ijms242015406

**Published:** 2023-10-20

**Authors:** Anna Fedorczak, Andrzej Lewiński, Renata Stawerska

**Affiliations:** 1 Department of Endocrinology and Metabolic Diseases, Polish Mother’s Memorial Hospital—Research Institute, 93-338 Lodz, Poland; anna.fedorczak@outlook.com (A.F.); andrzej.lewinski@umed.lodz.pl (A.L.); 2 Department of Endocrinology and Metabolic Diseases, Medical University of Lodz, 93-338 Lodz, Poland; 3 Department of Paediatric Endocrinology, Medical University of Lodz, 93-338 Lodz, Poland

**Keywords:** sirtuin 1, JAK2-STAT pathway, growth hormone, IGF-1, short stature children

## Abstract

The regulation of growth processes in children depends on the synthesis of growth hormone (GH) and insulin-like growth factor 1 (IGF-1). Insulin-like growth factor 1, which is mainly secreted in the liver in response to GH, is the main peripheral mediator of GH action. Newly discovered factors regulating GH secretion and its effects are being studied recently. One of them is sirtuin 1 (SIRT1). This NAD^+^-dependent deacetylase, by modulating the JAK2/STAT pathway, is involved in the transduction of the GH signal in hepatocytes, leading to the synthesis of IGF-1. In addition, it participates in the regulation of the synthesis of GHRH in the hypothalamus and GH in the somatotropic cells. SIRT1 is suggested to be involved in growth plate chondrogenesis and longitudinal bone growth as it has a positive effect on the epiphyseal growth plate. SIRT1 is also implicated in various cellular processes, including metabolism, cell cycle regulation, apoptosis, oxidative stress response, and DNA repair. Thus, its expression varies depending on the different metabolic states. During malnutrition, SIRT1 blocks GH signal transduction in hepatocytes to reduce the IGF-1 secretion and prevent hypoglycemia (i.e., it causes transient GH resistance). In this review, we focused on the influence of SIRT1 on GH signal transduction and the implications that may arise for growth processes in children.

## 1. Introduction

The regulation of growth processes in children depends on the synthesis, secretion, and action of growth hormone (GH) and insulin-like growth factor 1 (IGF-1). Insulin-like growth factor 1 IGF-1, which is mainly secreted in the liver in response to GH, is the main peripheral mediator of GH action. It is well known that GH is synthesized, stored, and secreted by somatotropic cells of the anterior pituitary gland. It is secreted in a circadian rhythm under the influence of the hypothalamic hormones: the stimulating effect of a GH-releasing hormone (GHRH, somatoliberin) and the inhibitory effect of a GH-inhibiting hormone (GHIH, somatostatin). GHRH and GHIH secretion is modulated by various hormones and factors [1,2]. One of them is ghrelin, which acts both indirectly, via the hypothalamus, where it modulates the release of the above mentioned hormones, and directly, via somatotrophs, where it stimulates GH secretion [3]. In addition, ghrelin also affects the orexigenic centre in the hypothalamus, stimulating food intake [4]. Somatoliberin, somatostatin, ghrelin, and their respective cascades of action, are important agents in the network of factors regulating GH secretion [5]. GH affects numerous cells, tissues, and organs, but its main mediator promoting longitudinal growth is IGF-1, synthesized mainly in the liver [6].

Recently, newly defined factors have been emerging that may contribute to the regulation of GH secretion and its effects, one of them being sirtuin 1 (SIRT1) [7,8]. This NAD+-dependent deacetylase, by modulating the Janus kinase 2 (JAK2)/Signal Transducers and Activators of Transcription (STAT) pathway, is involved in the transduction of the GH signal in hepatocytes, leading to the synthesis of IGF-1, but also in the regulation of GHRH synthesis in hypothalamic neurons with GH receptor activity [9,10]. It has been suggested that SIRT1 is involved in growth plate chondrogenesis and longitudinal bone growth [11]. It is also engaged in various cellular processes, including metabolism, cell cycle regulation, apoptosis, oxidative stress response, DNA repair, inflammation processes, as well as the regulation of hunger and satiety [12,13]. Thus, its expression changes according to different metabolic states to help maintain homeostasis. In this review, we have attempted to present the current knowledge concerning the involvement of SIRT1 in GH/IGF-1 signal transduction and to discuss its possible impact on growth processes in children.

## 2. GH Intracellular Signal Transduction—GHR-JAK2-STAT Pathway

The binding of GH to its cell surface receptor (GH receptor—GHR) leads to the activation of several intracellular signalling pathways. With respect to the growth processes of children, the main pathway is the JAK2/STAT5β pathway in the liver because it is responsible for IGF-1 synthesis [9].

The GHR is a member of the class 1 haematopoietic cytokine receptor family. Although this receptor is found on the cells of many tissues, the liver is the richest in it [10]. It is a cell surface glycoprotein receptor, and consists of a large extracellular domain containing the GH binding site, an intracellular domain and a single transmembrane domain (TD). The GH molecule binds on the cell surface to the binding site of one GH receptor and then to the other one. As a result, a complex is formed containing two GHRs (GHR dimer) in combination with one GH molecule. The dimerization of the GHR is critical for GHR signalling and GH action, because it results in a conformational change in GHR and—subsequently—the repositioning of the proximal part of the intracellular domain—the proline-rich region (Box1) associated with JAK2. This starts a JAK2-mediated tyrosine phosphorylation cascade involving JAK2 itself, the GHR and STATs [10]. The main STATs are 1, 3, and 5; the most important—for further consideration—are STAT3 and STAT5β. After their phosphorylation, STATs translocate from the cytoplasm to the nucleus and bind to the promoter region of DNA which results in the promotion the transcription of IGF-1.

Also, the IGF-2, IGF-binding protein 3 (IGFBP-3) and acid labile subunit (ALS) genes are promoted in the same mechanism [14]. This pathway is therefore responsible for the activation of the *IGF-1* gene and the synthesis of IGF-1, and also ensures the subsequent stability of IGF-1 in the serum, as it is involved in the production of the elements used to form the IGF-1–ALS–IGFBP-3 tertiary complex. It has been proven that SIRT1 is one of the modulators of the activity of the JAK2/STAT pathway [15].

The activation of JAK-STAT signalling occurs within minutes after GH stimulation, but is transient due to the tight regulation of the signalling termination by the suppressors of cytokine signalling (SOCS), protein tyrosine phosphatases (PTPs), protein inhibitors of activated states (PIAS), and GHR internalisation [10].

GH also activates other pathways, including the mitogen-activated protein kinase (MAPK), extracellular signal-regulated kinase (ERK) 1 and 2, as well as the insulin-receptor substrates (IRS) 1 and 2 and phosphatidyl inositol-3 kinase (PI3K) pathways. They clearly contribute to the metabolic effects of GH; however, their role in the growth of bones remains unclear [16,17,18]. Considering the cardinal importance of the GH–IGF-1 axis in linear human growth, the defects or modifications at various points of this axis may result in an impaired growth rate, leading to short stature [19,20].

## 3. IGF-1—The Main Mediator of GH Activity

It is well known that GH in peripheral tissues acts through IGF-1 [6]. IGF-1 is responsible for stimulating the growth of all cell types and causes significant metabolic effects [21,22]. As was mentioned above, IGF-1 is produced mainly in hepatocytes, but other cell types are also able to synthesise and secrete IGF-1 [6]. Besides GH, a number of other factors influence the synthesis of IGF-1, such as nutrients and nutritional status, some components of the immune system, as well as other hormones. IGF-1 inhibits hypothalamic GHRH release and directly inhibits GH production in pituitary somatotrophs by the negative feedback mechanism [6,22,23].

The IGF-1 bioavailability and stability in circulation depend on binding to specific proteins, IGFBP-3 in particular. IGFBPs both extend the half-life of IGFs and modulate IGFs’ availability and activity [24,25]. IGF-1/IGFBP-3 then binds ALS and thus forms a large (150 kDa) ternary complex, which is a reservoir of IGF-1 [6]. The IGF-1 and IGF-2 are liberated from this complex by pappalysin 2 (PAPPA2), which enables them to pass through the capillary epithelium and enter the interstitium. As it was mentioned above, IGF-2, IGFBP-3, and ALS synthesis and secretion are also dependent on the JAK2/STAT pathway and its modulator—SIRT1. In the target tissues, IGF-1 binds to two receptors: IGF-1R and IGF-2R, which exhibit intrinsic enzymatic activity. The growth-promoting effects of IGF-1 are mediated by IGF-1R, which is structurally homologous to the insulin receptor and can bind both IGF-1 and insulin, but has a greater affinity for IGF-1. In turn, IGF-1 can also bind to and activate the insulin receptor [5]. Thus, the effect of IGF-1 on glucose metabolism is similar to insulin (insulin-like effect) and—in contrast to GH—promotes hypoglycaemia. GH increases glucose production in the liver by enhancing glycogenolysis and gluconeogenesis [26,27], and in a state of energy deficiency, GH is an important signal for mobilizing body fat and glycogen in order to maintain normal blood glucose levels. This explains why a mechanism of GH resistance with respect to IGF-1 secretion can be triggered to maintain normal glucose levels and prevent hypoglycemia. There are many conditions in which IGF-1 secretion is reduced, even though GH secretion is normal or even elevated: e.g., liver disease, kidney dysfunction, sepsis, cancer, systemic autoimmune disease, conditions after severe surgical trauma, and other massive activations of the immune system (due to interleukin 1 and 6 activity) [28]. It has been proven that in patients with untreated celiac disease or other (even oligosymptomatic) gastrointestinal tract diseases, the concentration of IGF-1 is reduced, despite a normal GH secretion, confirmed in stimulating tests [29]. By the same mechanism, low IGF-1 levels with GH resistance are observed in malnourished children or children and adolescents with anorexia nervosa [30]. In all those cases, where these conditions are prolonged, the children’s growth rate slows down and improves after this unfavourable phenomenon disappears [29].

It seems that SIRT1, which regulates the JAK2/STAT pathway, may be involved in peripheral GH resistance and be responsible for the reduced IGF-1 concentration in some conditions (e.g., malnutrition) that require increased glucose production to prevent hypoglycaemia. In such cases, the rate of growth may be temporarily impaired.

## 4. SIRT1—The Key Player in Metabolism

**Figure 1:**
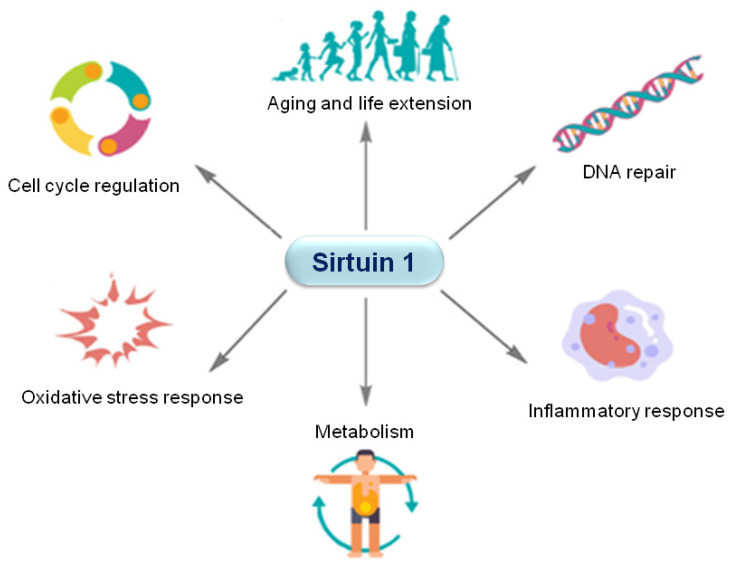
SIRT1 involvement in biological processes.

Sirtuins (silent information regulator 2 proteins) constitute a family of highly conserved NAD^+^-dependent deacetylases. They use NAD^+^ as an essential cofactor to remove acetyl groups from various proteins and alter their function. Seven types of sirtuins, SIRT 1–7, have been detected in mammals [7]. Sirtuins play key roles in responding to nutritional and environmental perturbations and are important in regulating a broad variety of cellular processes, e.g., metabolism, mitochondria homeostasis, autophagy, DNA repair, apoptosis, oxidative stress, and senescence [12,13,31]. Among the mammalian sirtuins, SIRT1 is the most extensively researched one. SIRT1 is the closest homolog to yeast Sir2p. It has been detected in multiple organs and tissues, including the liver, pancreas, brain, heart, muscle, and adipose tissue [32]. Although SIRT1 is mainly located in the nucleus, in certain cell types (e.g., pancreatic β cells) it can be localized in the cytoplasm [33]. SIRT1 interacts with and regulates a number of histone and non-histone protein substrates. Similar to other sirtuins, it is involved in cell cycle regulation, apoptosis, autophagy, oxidative stress response, DNA repair, and inflammation [32,34,35,36,37,38,39,40], as well as in the regulation of cellular senescence and aging [12,13,31,39] (Figure 1). Moreover, SIRT1 also modulates important cellular processes in the cardiovascular system [41]. SIRT1 acts in endothelial and smooth muscle cells to protect the vasculature [42,43,44].

**Figure 2:**
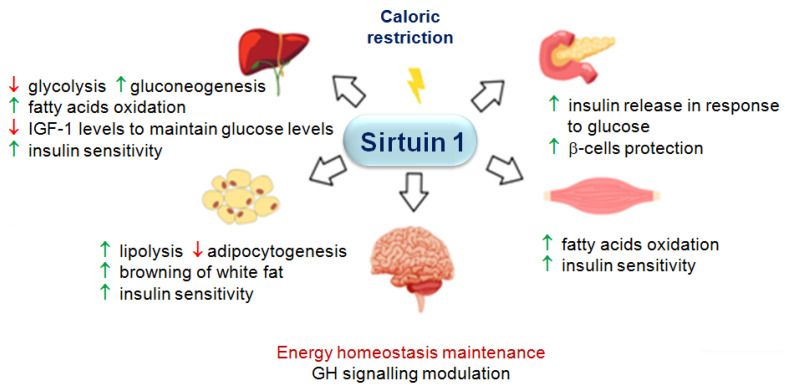
Summary of the effect of SIRT1 on various metabolic processes during caloric restriction. The red arrows indicates a reduction and the green arrows indicates an intensification of the processes in question.

The amount of SIRT1 depends on the availability and type of nutrients [45], because this nutrient-responsive protein is involved in responding to metabolic imbalances that are triggered by fasting, caloric restriction, and malnutrition. In a fasting state, caloric restriction, or malnutrition, it intensifies catabolic processes and inhibits anabolic processes to maintain homeostasis [32] (Figure 2). In the liver, SIRT1 promotes fatty acids’ oxidation via the activation of the peroxisome proliferator-activated receptor alpha (PPARα)/peroxisome proliferator-activated receptor-gamma coactivator (PGC)-1α signalling pathway [46], as well as inhibits fatty acids’ synthesis by attenuating the transcriptional activity of sterol regulatory element-binding protein (SREBP)-1c [47,48]. In skeletal muscles, SIRT1 correspondingly increases fatty acids’ oxidation via PGC-1α deacetylation [49,50]. In white adipose tissue, SIRT1 enhances lipolysis and fat mobilization from adipocytes, as well as reduces adipogenesis by inhibiting PPARγ [49,51]. SIRT1 drives white fat browning to disperse stored energy as heat [52]. Regarding glucose metabolism, SIRT1 stimulates gluconeogenesis in the liver by inhibiting STAT3, while activating PGC-1α and Forkhead box protein 1 (FOXO1), as well as inhibits glycolysis by deacetylating PGC-1α and by repressing the glycolytic enzyme PGAM-1 [53,54,55,56]. In pancreatic β-cells, SIRT1 enhances insulin release in response to glucose [33]. SIRT1 also regulates the production and secretion of insulin-sensitizing factors, such as adiponectin and fibroblast growth factor 21 (FGF21) through the regulation of FOXO1 and PPARγ [57]. Additionally, SIRT1 improves the insulin sensitivity of adipose tissue, skeletal muscle, and the liver [58]. What is particularly interesting is that SIRT1 inhibits GHR intracellular signal transduction for *IGF-1* gene expression to reduce IGF-1 synthesis and—in this way—maintains glucose levels [15,59].

## 5. Regulation of SIRT1 Activity

The classic activation of sirtuins is caused by an increase in the NAD+/NADH ratio. Thus, as mentioned, SIRT1 activation occurs under caloric restriction and starvation to enhance its catabolic effects. Also, physical activity increases the expression levels of SIRT1 [60]. However, there are other factors that regulate SIRT1 activity. A known SIRT1 activator is resveratrol—a polyphenol which can be found in red wine and some dietary products [61,62,63]. Quercetin, one of the major flavonoids which is widely expressed in fruits (grapes and peaches) and vegetables (onions and garlic) regulates cellular senescence and multiple aging-related cellular processes via SIRT1 [64]. The synthetic SIRT1-activating compounds (STACs), i.e., SRT1720, SRT2183, and SRT1460, have also been reported to activate SIRT1 in rodents and improve lipid profiles, glucose tolerance, and health span [12,65]. In turn, nicotinamide, a form of vitamin B3, was first introduced as a sirtuin inhibitor [66]. Several specific inhibitors of SIRT1 action have also been described, including sirtinol, cabol, tenovin-1 and tenovin-6, salermide, EX527, and others [67,68].

It seems that activation of SIRT1 by genetic or pharmacological methods can bring many metabolic benefits [69]. Sirtuin triggers are being considered for the treatment of obesity, and their beneficial effect could result from the intensification of catabolic processes [70,71,72].

Furthermore, it is important to mention the post-transcriptional factors that are involved in SIRT1 mRNA regulation. This post-transcriptional regulation is mediated by microRNAs (miRNAs) and RNA-binding proteins (RBPs) [42]. MicroRNAs are short non-coding RNAs that modulate target gene expression and, as a result, impact important processes including proliferation, apoptosis, and senescence. Various miRNAs have been proven to regulate SIRT1 expression and activity [42]. For example, miR-34a induces endothelial senescence and apoptosis through the inhibition of SIRT1 [73]. Whereas, RBPs are proteins that bind to RNA to form a ribonucleoprotein complex and play an important role in the post-transcriptional control of RNAs. Hu antigen R (HuR), one of the RBPs, promotes SIRT1 expression by directly binding to SIRT1 mRNA, as well as through indirect pathways via microRNA [74]. HuR and TIA1/TIAL1 are involved in the regulation of alternative splicing of SIRT1 pre-mRNA [75]. Moreover, adenosine deaminase, acting on RNA 1 (ADAR1), a RNA editing enzyme, regulates SIRT1 expression by affecting its RNA stability through HuR. Whereas, ADAR1 suppression promotes senescence via the downregulation of *SIRT1* mRNA by a decrease in HuR binding [76].

To sum up, the epigenetic regulation of SIRT1 may have impact on various pathways, concerning metabolic diseases, cardiovascular diseases, cancer, and senescence.

## 6. SIRT1 as a Negative Regulator of JAK2/STAT5β Pathway in the Liver and Its Impact on Growth

In a condition of caloric restriction, a number of adaptive processes to survive and provide energy for vital organs are activated. Most studies have shown that caloric restriction improves glucose metabolism, increases mitochondrial activity, and even extends life span [77]. In terms of growing, fasting induces GH resistance in the liver, leading to a decrease in the serum IGF-1 levels as one of the adaptive mechanisms for malnutrition [78]. Under fasting conditions, elevated GH levels are important to prevent hypoglycaemia and mobilize free fatty acids from adipose tissue, while reduced IGF-1 impairs growth and preserves energy for vital life processes [79]. It has been described that in poorly nourished children with short stature, despite increased ghrelin synthesis, low IGF-1 concentrations are observed [80].

As it has been explained above in detail, the GH intracellular signal transduction from the GH receptor leads through the JAK2/STAT5β pathway and results in IGF-1 synthesis. The GH signal transduction via the JAK2/STAT5β pathway may be impaired at different stages and due to various reasons. Besides the mutations that may occur in the *GHR*, *JAK2*, or *STAT5β* genes and result in a severe primary IGF-1 deficiency and short stature [81,82,83], there are some malfunctions of the GHR-JAK2-STAT pathway which may be caused by many modulators, some of which may be linked to eating habits or result from the action of other modifiable factors, one of them being SIRT1 [5]. Yamamoto et al. [15] proved SIRT1’s role in that adaptation, concluding that SIRT1 regulates GH-induced IGF-1 production in the liver, thereby reducing the serum concentration of IGF-1. Also, SIRT1 negatively regulates GH-induced IGF-1 mRNA production, which was confirmed in a human hepatocellular carcinoma cell line (HepG2) and rat primary hepatocytes, with the use of SIRT1 inhibitors (sirtinol and nicotinamide) and stimulators (resveratrol and NAD) [15].

In the fed condition, the Src Homology 2 (SH2) domain of STAT5 recognizes and binds to tyr-phosphorylated GHR, causing JAK2 to phosphorylate and activate STAT5. In the fasting condition, activated SIRT1 interacts with STAT5 by deacetylating the lys residues adjacent to the SH2 domain of STAT5. As a result, the activation of STAT5 is inhibited by the impaired ability to bind tyr-phosphorylated GHR. In turn, fasting-induced GH resistance, as well as tyr-phosphorylation of STAT5 are restored by treatment with a SIRT1 inhibitor—nicotinamide [15].

SIRT1 also exerts an inhibitory effect on STAT3 activity [53]. It was demonstrated that treating the immobilized mouse hepatocytes of a SV40 cell line with nicotinamide increased the level of acetylation and phosphorylation of STAT3, and its activity was independent of JAK2 [53]. The levels of STAT3 acetylation and phosphorylation are constitutively higher in the SIRT1 knockout mice embryonic fibroblasts (MEFs) than in the wild-type ones. The cell culture treatment with a SIRT1 inhibitor (EX527) increases, while SIRT1 activator (resveratrol) decreases, STAT3 acetylation and phosphorylation only in wild-type MEFs, but not in SIRT1 knockout mice MEFs. Thus, deacetylation of STAT3 also depends on SIRT1 [53].

Summing up, it has been found that under fasting conditions, SIRT1 can inhibit GH signalling by interacting with STAT3 and/or STAT5, and in this way negatively regulates GH-induced IGF-1 production. SIRT1-dependent negative regulation of GH-induced IGF-1 production seems to be an adaptive mechanism under fasting conditions and malnutrition. However, the role of SIRT1 in the regulation of the GH–IGF-1 axis occurs not only at the level of the liver, but also in the central nervous system.

## 7. SIRT1 in the Hypothalamic–Pituitary Axis

In addition to the metabolic functions of SIRT1 discussed above and its involvement in GH-induced IGF-1 synthesis in hepatocytes, SIRT1 also plays an important role in the regulatory mechanisms in the hypothalamus and in the hypothalamic–pituitary axis [8].

SIRT1 mRNA is widely expressed throughout the central nervous system. It shows nuclear localization in the neurons of the hypothalamus, hippocampus, and extranuclear localization in the neurons of the dorsal regions of cerebral cortex. In the hypothalamus, SIRT1 is expressed in the arcuate (ARC), ventromedial (VMH), dorsomedial (DMH), lateral (LH), and paraventricular (PVN) nuclei, brain regions that all play critical roles in the central regulation of the adaptive responses to food availability, energy expenditure, thermoregulation, as well as the synthesis of GHRH and GHIH. SIRT1 is also expressed in the suprachiasmatic nucleus (SCN), a region important for the central regulation of circadian rhythms [84,85].

For over a dozen years, intensive research has been conducted (mainly on an animal model) on the role of SIRT1 in the regulation of the hunger and satiety centres, as well as on the impact of its deficiency and excess in this region on behaviour regarding food intake and physical activity, as well as on energy expenditure. As a result of these studies, SIRT1 appears to mediate several processes controlled by the hypothalamus. Caloric restriction and fasting have been found to increase SIRT1 expression and activity, not only in hepatocytes but also in the hypothalamus [84,85,86,87]. It has already been proven that SIRT1’s effects on the energy balance are mediated through melanocortin signalling. The hypothalamic part of the central melanocortin system is located in the ARC and engaged in the regulation of food intake, energy expenditure, and metabolism [88]. SIRT1 regulates the expression of both agouti-related protein (AgRP) and proopiomelanocortin (POMC) neurons, which produce neuropeptides that are responsible for appetite stimulation (the oryxogenic effect) and food intake reduction (anorexigenic effect), respectively [86,87,89,90,91]. During fasting, SIRT1 induces food intake (orexigenic effect via AgRP activity). The inhibition of hypothalamic SIRT1 activity resulted in decreased AgRP and increased POMC expressions [86,90]. In the feeding state, SIRT1 decreased the expression of the orexigenic neuropeptide agouti-related peptide and reduced food intake [87].

The overexpression of SIRT1 in POMC neurons was associated with increased sympathetic activity in adipose tissue, leading to increased energy expenditure and a lean phenotype [89]. Surprisingly, the overexpression of SIRT1 in AgRP neurons also suppressed food intake [85,89].

SIRT1 also plays an important role in the neurobehavioral adaptation to energy limitation and in maintaining homeostasis. The overexpression of brain-specific SIRT1 in transgenic mice resulted in the increased activation of neurons in the DMH and LH, maintenance of a higher body temperature range, and increased physical activity in response to various dietary restrictive conditions [92]. In contrast, SIRT1-deficient mice exhibited defects in neurobehavioral adaptation to diet-restricting conditions [85].

It seems that hypothalamic SIRT1 acts as a modulator of GH signalling. SIRT1 brain-specific knockout mice were dwarfed and had reduced somatotropic signalling—both GH and IGF-1 were decreased [93]. Although the central SIRT1/p53 pathway mediates the orexigenic action of ghrelin, blocking this pathway does not modify ghrelin-induced GH secretion [94]. The injection of a SIRT1 inhibitor blunted the effect of ghrelin on food intake, whereas it did not change ghrelin-induced increase in plasma GH levels [94]. This data suggest that at the central level, SIRT1 may play a role in the regulation of GH signalling in other mechanisms.

Growth hormone modulates the neuroendocrine responses to food deprivation via AgRP neurons [95]. AgRP-specific GHR ablation mitigates the fasting-induced activation of AgRP/NPY neurons and neuroendocrine energy-saving adaptations to caloric restriction [95]. SIRT1 expression is present in the AgRP neurons that express GHR [96]. In the fasted state, SIRT1 expression increases in AgRP neurons, whereas, in animals with GHR deletion in AgRP neurons, this response is attenuated. Thus, SIRT1 appears to act as a hypothalamic mediator of the GHR signaling in the adaptive responses to fasting [96].

SIRT1 also mediates the effects of the supraphysiological GH levels. SIRT1 levels were elevated not only in caloric-restricted mice but also in mice overexpressing GH. However, in GH receptor knockout (GHRKO) mice, despite low IGF-1, the SIRT1 protein levels were not increased [97].

On the other hand, SIRT1 has been shown to inhibit the transcription factor cAMP response element-binding protein (CREB) and negatively regulate pituitary GH synthesis [98].

In conclusion, the presented data indicate that the region-specific expression of SIRT1 plays a significant role in the regulation of appetite, energy expenditure, general metabolism, and growth. SIRT1 has been found to modulate the hypothalamus–pituitary axis.

## 8. SIRT1 in IUGR and in Short Stature Children

The negative relation between SIRT1 and IGF-1 in the context of growth was observed in intrauterine growth restriction (IUGR) cases. Chriett et al. [99] examined the impact of IUGR on hormones and gene expression in pig skeletal muscle. The gene expression trajectories of the sirtuins and metabolic genes were altered in IUGR and correlated to IGF-1 dysregulation. Higher *SIRT1* gene expression and lower IGF-1 levels were observed in the pig IUGR group [99]. The catch-up growth model in children born small for gestational age (SGA) has been discussed by Griffin [59]. If the fetus is malnourished during pregnancy, the secretion of ghrelin increases, which stimulates the hunger centre, as well as the secretion of GH. This should increase the IGF-1 pool. However, at the same time, caloric restriction induces the synthesis of SIRT1 and FGF21, which block GH signal transduction in the liver by obstructing the JAK/STAT pathway, thus limiting IGF-1 production. Thus, despite a high level of GH, intrauterine growth retardation is observed. Postpartum, as nutrients become available, the SIRT1 and FGF21 levels decline, the liver sensitivity to GH returns to normal, and IGF-1 production resumes. This thesis is confirmed by the fact that in prepubertal children with SGA who have undergone the catch-up phenomenon (with currently normal height), the concentrations of both ghrelin and IGF-1 are significantly higher than in children with SGA without the catch-up phenomenon (with permanent short stature) [100].

So far, there have been few studies on the SIRT1 levels in short stature children. Interestingly, in the study performed by Kaplan et al. [101] on short stature children, both the SIRT1 and IGF-1 serum levels were decreased in the untreated with GH short stature children compared to the GH treatment children. However, it is not clear whether among the children with short stature there were children with GHD or ISS, and the BMI SDS was lower in the study groups.

It should also be taken into account that the serum levels of SIRT1 may not reflect its activity in different tissues and organs. One of these tissues is the epiphyseal growth plate, because the effect of SIRT1 on the expression and production of IGF-1 in chondrocytes will be important for the longitudinal growth of children.

## 9. SIRT1 Activity in Chondrocytes

Chondrocytes in the growth plate are influenced by various regulatory factors and hormones that together determine the rate of proliferation and maturation until late puberty when the growth plate fuses [102]. Among many others (i.e., estrogens, androgens, or PTHrP), both the GH receptors and the IGF-1 receptors are expressed on human growth plate chondrocytes [86]. The data suggest that SIRT1 may have a positive impact on the growth plate in chondrocytes. Gabay et al. [103] established that SIRT1 enzymatic activity is needed for cartilage homeostasis—they demonstrated that mice with defective SIRT1 also had defective cartilage, with elevated rates of cartilage degradation with age [103]. Shtaif et al. investigated the role of SIRT1 in modulating the response of the epiphyseal growth plate to nutritional manipulation [11]. In collagen type II-specific SIRT1 knockout mice the epiphyseal growth plate was less organized and catch-up growth was less efficient [11]. SIRT1 appears to be important in the proper regulation of chondrogenesis. In the study presented by Jin et al., SIRT1 ablation inhibited growth plate chondrogenesis and contributed to growth retardation through the hyperactivation of mammalian target of rapamycin complex 1 (mTORC1) signaling [104]. Whereas, the findings reported by Kang et al. indicate that SIRT1 deacetylates protein kinase-like endoplasmic reticulum kinase (PERK) and attenuates the PERK–Eukaryotic Initiation Factor 2 (eIF-2)–C/EBP-homologous protein (CHOP) pathway in chondrocytes and thus promotes growth plate chondrogenesis and longitudinal bone growth [105]. Authors confirmed that SIRT1 expression in growth plate facilitates chondrocyte proliferation and hypertrophy as well as prevents apoptosis [104,105]. Conversely, it was found that DNA damage-inducible transcript 3 (DDIT3)/CHOP interfere with SIRT1 to stimulate autophagy [106] as well as SIRT1 directly triggers autophagy in chondrocytes [107]. Moreover, in the condition of endoplasmic reticulum stress, DDIT3/CHOP upregulated SIRT1 and thereby had an inhibitory effect on chondrocyte differentiation and matrix synthesis.

Interestingly, studies have demonstrated the direct link between epiphyseal growth plate micro RNA and SIRT1. Cheng et al. [108] showed that SIRT1 exerts a protective effect on growth plate chondrocytes under dexamethasone stimulation. MiR-211-5p downregulated SIRT1 in growth plate chondrocytes treated with dexamethasone. The inhibition of miR-211-5p led to an elevation in SIRT1 expression, subsequently restoring the function of chondrocytes [108]. Whereas, Pando et al. proved that nutrition restriction decreased miR-140 levels leading to the elevation of SIRT1 [109]. Authors have concluded that SIRT1 upregulation may inactivate hypoxia inducible factor 1α (HIF-1α), which is necessary for chondrocyte survival [109,110,111].

Overall, the data suggest that SIRT1 is involved in growth plate chondrogenesis and longitudinal bone growth as well as chondrocyte homeostasis; however, the exact mechanisms responsible for this process remain to be clarified.

## 10. Conclusions

In conclusion, SIRT1 is an important factor in the regulation of various biological processes, with particular emphasis on maintaining homeostasis. This review indicates that SIRT1 may modulate growth processes in children, acting differently depending on the cell type and conditions in a tissue- and context-specific manner (Table 1).

It seems that SIRT1 modifies the GH/IGF-1 axis by affecting the transmission of signals in the hypothalamic neurons releasing GHRH, AgRP, and POMC, as well as by modulating ghrelin action. It promotes the secretion of GH from the pituitary, but during caloric restriction, it inhibits the peripheral action of GH by decreasing the expression and secretion of IGF-1. The purpose of this modulation is to maintain a balance between stimulating and inhibiting growth processes, preserving the energy necessary for important metabolic processes in maintaining homeostasis.

Thus, it appears that in children with malnutrition, the level of SIRT1 is increased to inhibit peripheral IGF-1 production and maintain homeostasis. This, in turn, slows down the growth rate of children and inhibits the maturation and ossification of their epiphyseal cartilages. This mechanism allows for a potential improvement in the final height if the current unfavourable conditions are improved. Considering that SIRT1 activity can be modulated by various factors, it should be noted that sirtuin triggers and inhibitors may be utilized in the future to develop new treatments for children with growth disorders.

## 11. Methodology 

We conducted a search of the literature from reputable databases, including PubMed and Google Scholar. Articles published up to December 2022 were taken into consideration, with later updates in September 2023. The search was limited to English-language articles. Combinations of the following MeSH terms were searched: growth, children, growth hormone, GH, sirtuin 1, SIRT1, sirtuins, insulin-growth like factor 1, IGF-1, STAT5, hypothalamus, hepatocytes, liver, chondrocytes, caloric restriction, fasting, and malnutrition. The initial search was followed by the removal of duplicate articles. The whole paper was read only if the abstract indicated relevant content. We incorporated peer-reviewed articles that directly addressed our research topic. In addition, a reference search was performed to find other important manuscripts. Finally, 111 articles were included in this review. Findings were summarized and discussed in the subsequent sections of this review.

## Figures and Tables

**Table 1:** SIRT1 actions with respect to growth and food supply.

Condition	AgRP Neurons	POMC Neurons	GHR Neurons	Hepatocytes
Feeding	Stabilized SIRT1 expression → decreased AgRP activity → decreased food intake	SIRT1 activity in POMC neurons is required for normal energy expenditure adaptations	SIRT1 is needed for GH synthesis or secretion	Preserved GH signalling
Caloric restriction	High SIRT1 expression → increased AgRP activity → increased food intake	Decreased POMC activity → reduced energy expenditure	High SIRT1 expression → stimulation to increase GH synthesis (for hyperglycaemic and other metabolic effects)	High SIRT1 expression → inhibition of GH signal transduction to decrease IGF-1 synthesis (to reduce hypoglycaemic and growth effects)
Knock-out/inhibition of SIRT1	Decreased AgRP activity → decreased food intake	Increased POMC activity → reduced food intake, increased energy expenditure	Impaired GH signalling → decreased GH and IGF-1	Enhanced GH-induced increase in serum IGF-1
Overexpression of SIRT1	Food intake suppression	Increased energy expenditure	Increased GH synthesis?	Suppressed GH-induced IGF-1 production

## Data Availability

Not applicable.
